# Developmental and epileptic encephalopathies after successful treatment of pediatric ALL: A case series and review of literature

**DOI:** 10.1002/epd2.20280

**Published:** 2024-09-11

**Authors:** Vanita Shukla, Sylvia Cheng, Juliette Hukin, Linda Huh, Anita N. Datta

**Affiliations:** ^1^ Department of Pediatrics, Division of Neurology, Faculty of Medicine, BC Children's Hospital University of British Columbia Vancouver Vancouver British Columbia Canada; ^2^ Department of Hematology, Oncology, BMT, Faculty of Medicine University of British Columbia Vancouver British Columbia Canada

**Keywords:** epileptic encephalopathy (not otherwise classified), Lennox–Gastaut syndrome, spasms (epileptic), tonic seizure: atonic seizure

## Abstract

Successful treatment of acute lymphoblastic leukemia (ALL) requires multiagent chemotherapy regimens and central nervous system prophylaxis, including intrathecal methotrexate. Although acute symptomatic seizures can occur during ALL treatment, epilepsy is less common. Furthermore, drug resistant epilepsy (DRE) is rare, presenting with two phenotypes: focal epilepsy, such as temporal lobe, or epileptic encephalopathies (EE), such as Lennox–Gastaut syndrome (LGS). For ALL survivors, the development of DRE has significant impact on morbidity, mortality, and quality of life. We describe four patients with ALL remission, who developed EEs, of which 3 had LGS. Mean age at ALL diagnosis was 1.9 years; range 1.1–2.5 years. All, but one, had normal development prior to ALL. No patient had CNS leukemic involvement. All patients received CNS prophylaxis with intrathecal methotrexate, without cranial radiotherapy. Three had symptomatic methotrexate neurotoxicity during treatment. The mean age at first seizure was 5.6 years; range 3.9–7.5 years, with a mean latency of 3.7 years from ALL diagnosis. All patients developed drug resistant EEs, moderate intellectual disability, and neuropsychiatric co‐morbidities. Two patients had a minimal response to corpus callosotomy (CC), and one did not respond the ketogenic diet. Successful treatment of childhood ALL is rarely associated with the development of DRE and EEs. Young age at ALL diagnosis (<3 years) may be a predisposing factor. Palliative treatments, including ketogenic diet and CC have limited benefit in these patients. Individual genetic susceptibility to MTX toxicity is likely related to epileptogenesis, and further research is required for epilepsy biomarkers.

## 1 INTRODUCTION

Acute lymphoblastic leukemia (ALL) is the most common childhood cancer, comprising 25% of cancer cases in those <15 years of age.^1^ Multiagent chemotherapy regimens and central nervous system (CNS) prophylaxis enable >85.8% survival rates.^1^ Seizures occur in 10%–13% of patients around chemotherapy induction.^2^ A small proportion of ALL patients develop epilepsy unrelated to acute CNS complications or leukemic involvement, although prevalence data is limited.^3^ Even rarer is drug‐resistant epilepsy (DRE), with two phenotypes: focal epilepsy, such as temporal lobe^4^ and epileptic encephalopathy (EE), where cognition is impacted beyond what is expected from the underlying pathology alone, such as methotrexate (MTX) neurotoxicity.^5^, ^6^, ^7^, ^8^, ^9^, ^10^, ^11^, ^12^ From 2000 to 2015, four ALL remission patients with EEs were identified at one tertiary care institution, confirming that this is rare, and their clinical courses are described.

**FIGURE 1:**
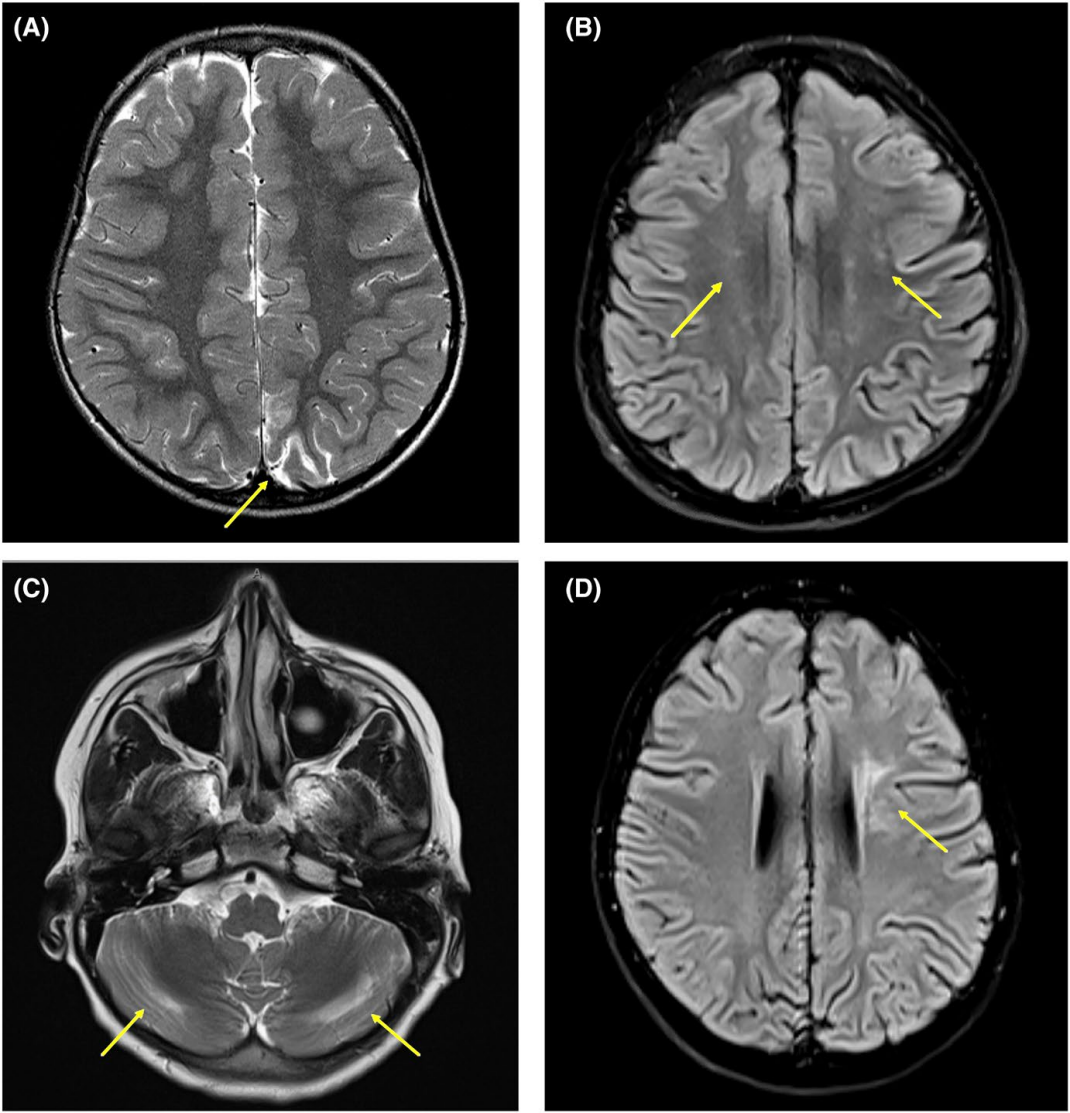
Neuroimaging Findings (Findings depicted by arrows). (A) Case 1: Axial T2. Focal cortical tissue loss at the posteromedial left cerebral hemisphere centered on the parieto‐occipital sulcus. (B) Case 2: Coronal FLAIR. Patchy FLAIR hyperintensities in the subcortical white matter. (C) Case 3: Axial T2. ↑T2 intensities in bilateral cerebellar hemispheres. (D) Case 4: Axial FLAIR. Increased FLAIR signal of the left frontal periventricular subcortical white matter. Cases 3 and 4 had symptomatic MTX neurotoxicity and increased FLAIR signals may reflect residual MTX neurotoxicity. Increased white matter intensities in Case 2 may reflect residual asymptomatic MTX neurotoxicity.

**FIGURE 2:**
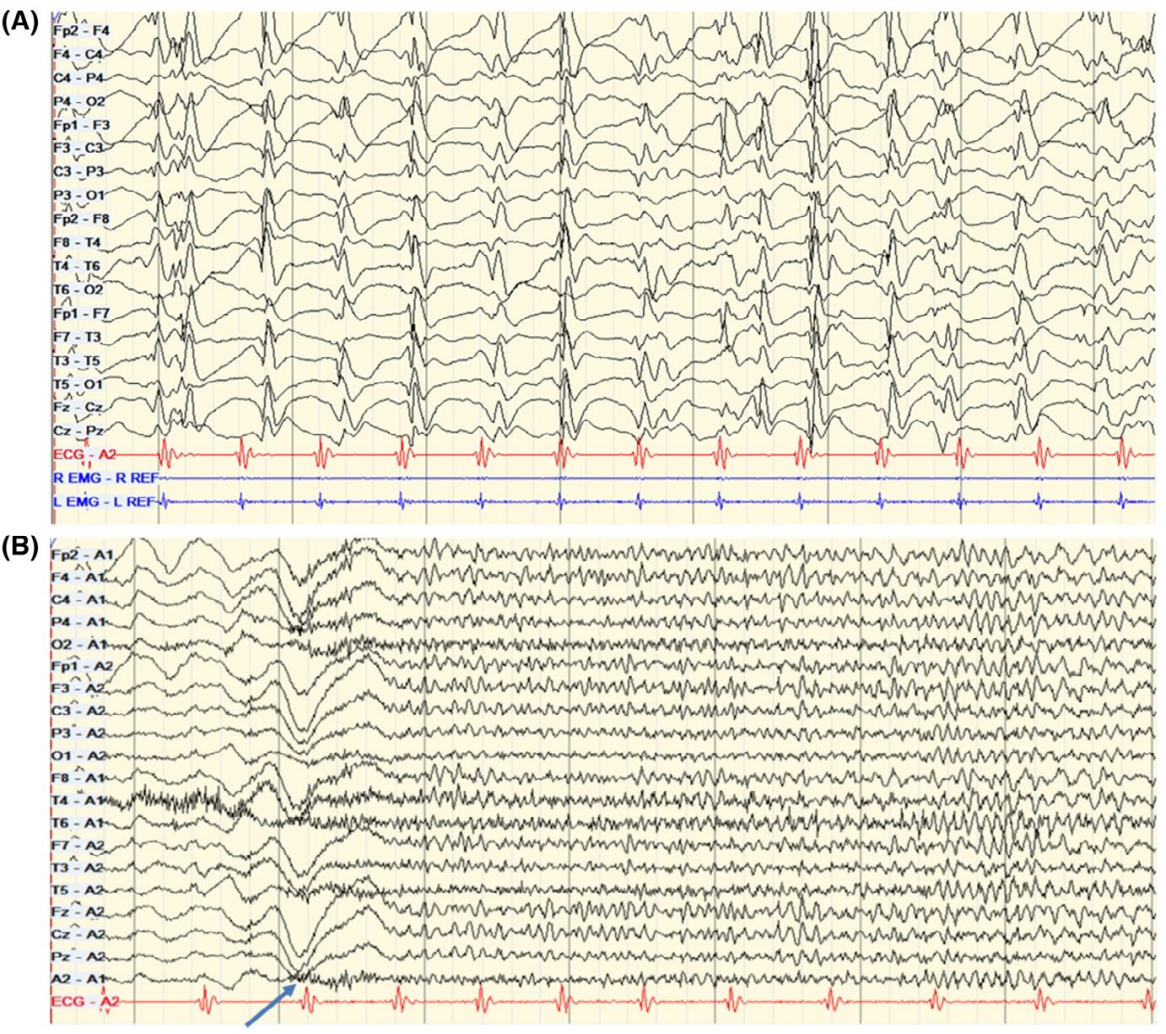
Interictal and Ictal EEG samples (Case 4). (A) Interictal EEG during quiet sleep demonstrates generalized slow spike‐wave discharges. The background is suppressed, and no sleep architecture is present. Bipolar montage; LFF: 1 Hz; HFF: 70 Hz; Time base 30 mm/s; Sensitivity 30microV/mm. (B) Ictal EEG demonstrates an electro‐decremental response associated with a clinical tonic seizure at the time indicated by the arrow. Referential montage (A1, A2); LFF 1 Hz; HFF 70 Hz; Time base 30 mm/s; Sensitivity 30 microV/mm.

Clinical data were collected via chart review (Table 1). The mean age at ALL diagnosis was 1.9 years (range 1.1–2.5 years), and all but one had normal prior development. None had CNS leukemic involvement. Patients followed chemotherapy protocols (Figure S1) with CNS prophylaxis using intrathecal (IT) MTX, without cranial radiotherapy. Three developed symptomatic MTX neurotoxicity. Other causes for epilepsy and acute symptomatic seizures were excluded. All achieved ALL remission. The mean age at first seizure was 5.6 years (range 3.9–7.5 years), with a mean latency of 3.7 years from diagnosis. All had neuroimaging abnormalities, EE, intellectual disability, and disabling neuropsychiatric co‐morbidities. Genetic testing (whole exome sequencing and chromosomal microarray) revealed no epilepsy‐related findings (Figures 1 and 2).

**TABLE 1:** Clinical features of 4 patients with ALL remission and DEE.

Case	Sex	Epilepsy Syndrome	ALL (Y)	Sz onset (Y)	ALL Rx protocol how MTX administered^a^	MTX NT: 1. Present 2. Sx 3. Time of NT from ALL Rx onset (Y) 4. Neuro‐imaging	Sz types	EEG: 1. BG 2. EDs 3. Sleep architecture	MRI	# ASMs tried	Other Rx	Age last follow‐up (Y)	1.Development/FSIQ^b^ 2. Co‐morbidities 3. School support 4. ADL support (yes/No)	Sz outcome	Genetic testing abnormalities (WES and CMA)
1	F	Multifocal/	1.9	3.9	CCG1961	1. Yes	FT, GTC, FIA	1. Mild slowing	Mild diffuse atrophy; focal atrophy left parietal‐occipital sulcus	10	‐	24	1. Moderate ID/FSIQ: 46	Sz‐free for 7 years (3 ASMs)	CMA: 14q32.33 duplication
		Generalized DEE			IT, Oral MTX	2. SE		2. Frequent MF (max left frontal); GSW					2. Anxiety, SIB		16p11.2 deletion
						3. 1.1 months		3. Present					3. Full time EA, IEP		
						4. CT: Bilateral PV white matter changes							4. Yes		
2	M	LGS	1.1	4.5	AALL0331	No	T, A, GTC, ES	1.Slow; dysrhythmic; PFA	Mild diffuse atrophy; patchy ↑T2 in SC WM	11	KD; CC	14	1. Moderate ID/no FSIQ^c^ (very low range)	Daily ES and T, (less lntense since CC)	Normal
					IT, IV, Oral MTX			2.Generalized SSS; MF					2. ASD		
								3. Absent					3. Full time EA, IEP		
													4. Yes		
3	M	LGS	2	7.5	AALL0232	1. Yes	T, ES	1.Dysrhythmc; slow; PFA	Mild diffuse atrophy; ↑T2 in cerebellar hemispheres	4	‐	12	1. Moderate ID/FSIQ: 55	Daily T	WES: Heterozygous c.4123G > A, p.(Glu1375Lys)CHD2 missense variant [NM001271.3]: VUS
					IT, IV, Oral MTX	2. ACS		2. Generalized SSS; MF					2. ADHD, ODD, OCD		
						3. 1		3.Absent					3. Full time EA, IEP.		
						4. MRI: patchy ↑T2 PV SC WM							4. Yes		
4	M	LGS	2.5	6.5	AALL0932	1. Yes	AA, T, A	1.Slow; discontinous in sleep.	Mild diffuse atrophy	8	CC	10	1. Moderate ID/FSIQ: 54	Daily T, A. (Less intense since CC)	Normal
					IT, IV, Oral MTX	2. ACS		2.Generalized SSS	↑T2 left frontal SC WM				2. ADHD, aggression		
						3. 0.8		3. Absent					3. Full time EA, IEP.		
						4. MRI: ↑T2 SC WM frontal, parietal, posterior temporal							4. Yes		

Abbreviations: #, number; ↑T2, Increased T2 MRI signal; A, atonic; AA, atypical absence; ACS, acute confusional state; ADHD, attention‐deficit‐hyperactivity disorder; ADL, activities of daily living; ALL, acute lymphoblastic leukemia; ASD, autism spectrum disorder; ASM, anti‐seizure medication; BG, background; CC, corpus callosotomy; CMA, chromosomal microarray; DEE, developmental and epileptic encephalopathy; Dx, diagnosis; EA, educational assistant; ED, epileptiform discharges; ES, epileptic spasm; F, female; FIA, focal impaired aware; FSIQ, full‐scale intelligence quotient; FT, focal tonic; GSW, generalized spike wave; GTC, generalized tonic–clonic; ID, intellectual disability; IEP, individualized education plan; IT, intrathecal; KD, ketogenic diet; LGS, Lennox–Gastaut Syndrome; M, male; MF, multifocal; MTX, methotrexate; NT, neurotoxicity (symptomatic); OCD, obsessive‐compulsive disorder; ODD, oppositional defiant disorder; PFA, paroxysmal fast activity; PV, periventricular; Rx, treatment; SC, subcortical; SE, status epilepticus; SIB, self‐injurious behavior; SSS, slow spike wave; Sx, symptoms; Sz, seizure; T, tonic seizure; VCR, vincristine; WES, whole exome sequecing; WM, white matter; Y, year.

^a^ North American Children's Cancer Group (CCG) and Children's Oncology Group (COG) treatment protocols. Treatment Protocols for ALL outlined in Figure S1.

^b^ Development is based according to the criteria in the Diagnostic and Statistical Manuel of Mental Disorders, Fifth Edition (DSM‐5). Neuropsychological assessments and FSIQ were determined after the diagnosis of epilepsy.

^c^ Case 2 could not fully cooperate with all administered testing, but intellectual abilities are documented as very low range.

## 2 CASE STUDIES

### 2.1 Case 1

A 24‐year‐old woman experienced simple febrile seizures at 6 months of age and was diagnosed with ALL at 23 months of age. Due to an elevated white cell count and a mediastinal mass, she was treated with CCG1961 high‐risk protocol, completing therapy at age 5 years. At age 3 years, she had status epilepticus from MTX neurotoxicity. Later that year, she developed focal tonic, bilateral tonic–clonic and focal impaired awareness seizures. EEG revealed a slow background and frequent generalized and multi‐focal epileptiform discharges, which were most active in the left frontal region. MRI of the brain demonstrated focal atrophy adjacent to the left parieto‐occipital sulcus. She tried 10 ASMs and has been seizure‐free since age 15 years. She remains on 3 ASMs due to persistently abnormal EEG. She attended a modified school, lives in a group home, and needs help with daily activities. She also has disabling self‐injurious behavior and anxiety.

### 2.2 Case 2

A 14‐year‐old male was diagnosed with B‐lineage ALL at 13 months of age. He had mild gross motor delay prior to diagnosis. He was treated with an ultra‐high‐risk protocol, AALL0331, based on bone marrow results. At age 3.5 years, he completed therapy. At age 4.5 years of age, he developed tonic, atonic, generalized tonic–clonic seizures and epileptic spasms. He tried a total of 14 ASMs and the ketogenic diet (4.5:1 ratio), with minimal response. At age 11 years, he underwent an anterior 2/3 corpus callosotomy (CC), resulting in shorter clusters of epileptic spasms. Tonic seizures stopped for 1 year but resumed to daily frequency. EEG showed slow spike–wave discharges (<3 Hz), sleep‐related paroxysmal fast activity and multiple seizure types, consistent with Lennox–Gastaut Syndrome (LGS). MRI showed diffuse atrophy and patchy increased T2 signal in the subcortical white matter. He attends school part‐time, with an individual education plan (IEP) and full‐time educational assistant (EA). He is diagnosed with ASD.

### 2.3 Case 3

A 12‐year‐old boy, previously healthy, was diagnosed with B‐cell ALL at 2 years of age. He was treated as per high‐risk AALL0232 protocol, completing treatment at 6 years of age. A year into treatment, he developed acute confusion from MTX neurotoxicity. By age 7.5, he had tonic seizures and epileptic spasms, with an EEG indicating LGS. MRI showed increased signals in the cerebellar hemispheres and atrophy at the junction of the posterolateral cerebellar hemispheres. He has an IEP and a full‐time EA at school. He is diagnosed with attention‐deficit disorder, obsessive‐compulsive disorder, and oppositional defiant disorder. He has social difficulties, including exhibitionism, and aggressive behaviors.

### 2.4 Case 4

An 11‐year‐old boy was diagnosed with pre‐B‐cell ALL at 2.5 years of age. He was treated with protocol AALL0932, as standard risk, completing therapy at age 5 years. During his first year of treatment, he experienced acute confusion from MTX neurotoxicity. By 6.5 years of age, he developed atypical absence, tonic, and atonic seizures, with an EEG showing LGS and an MRI indicating mild diffuse atrophy and increased T2 signal in the left periventricular white matter. After trying 10 ASMs, he underwent an anterior 2/3 CC at age 9 years due to disabling atonic seizures, which reduced seizure intensity but not frequency. He has an IEP and full‐time EA at school. Extreme aggressive behaviors are managed by neuropsychiatry.

## 3 DISCUSSION

In this series, 4 patients in ALL remission developed EEs, with substantial impact on quality of life. Previous reports, excluding other causes of epilepsy and acute symptomatic seizures, describe ALL patients with drug‐resistant EEs (Table 2).^5^, ^6^, ^7^, ^8^, ^9^, ^10^, ^11^, ^12^ Although 2 previous reports describe EEs associated with leukemic CNS involvement,^5^, ^11^ this was not the case in our series, or other reports,^6^, ^7^, ^8^, ^9^, ^10^, ^12^ indicating that MTX neurotoxicity is likely a primary cause of epileptogenesis.

**TABLE 2:** Literature review: patients with ALL remission and drug‐resistant EE (multifocal‐generalized).

Study (year) [reference]	Case/sex	DEE type	Sz onset (Y)	ALL (Y)	Documented symptomatic MTX neurotoxicity (+)	ALL Rx	CNS ALL (+)	EEG (background and epileptfiorm discharges)	MRI brain	Other epilepsy Rx	Outcome
Abushanab (2021)^6^	1/M	LGS	4	2	+	Sys, IT		Slow BG; SSW, PSW	Normal	CC	Daily sz (no response to CC); Significant ID
										VNS	
	2/F	LGS	8	3		Sys, IT	+	Slow BG; SSW, PSW	Normal	CC	Cessation of T; Other sz weekly/monthly
										VNS	
	3/M	LGS	6.5	2		Sys, IT		Slow BG; SSW, PSW	Normal	VNS	Lost to follow‐up
										CC	
	4/M	LGS	5	2.5		Sys, IT		Slow BG; SSW, PSW	Normal	−	Frequent sz; Significant ID
Smith (2018)^8^	5/F	LGS	9	−		Sys, IT		Slow BG, PFA; GSW/PSW	Diffuse atrophy, colpocephaly	−	Daily
						RT					sz, signficiant ID, behavioral concerns
	6/F	LGS	11	−		Sys, IT		Slow BG, PFA; GSW/PSW	Single right periventricula rnodular heterotopia +	VNS	Persistent A, significant ID
									Transmantle		
									Dysplasia		
Gonzalez‐Otarula (2016)^7^	7/F	LGS	5	1.5		Sys, IT		Slow BG; SSW	Normal	CC	Daily sz, Moderate ID
						RT				VNS, KD	
	8/F	LGS	3.5	2.5		Sys, IT		Slow BG; SSW, PSW	Cortical and subcortical calcifications	VNS	Persistent AA, Moderate ID
	9/F	DEE (other)	10	4		Sys, IT		BG normal; MF, GSW	Normal	VNS offered	Daily AA; Mild ID
Fasano (2009)^5^	10/M	DEE (other)	15	2.5		Sys, IT		No BG description; F, MF, GSW	Normal	−	6 sz/year; ID (espcially verbal); behavioral concerns
						RT					
	11/F	LGS	9	3		Sys, IT		NO BG description; MF, GSW	↑T2 Left mesial temporal	VNS	Daily sz, significant ID
						RT					
	12/M	LGS	9	0.7		Sys, IT		No BG descrioption; GSW; F (bifrontal)	↑T2, FLAIR, periventricular white matter	KD	Daily sz; significant ID
						RT					
Khan (2003)^9^	13/F	LGS	1.2	0.2		Sys, IT		BG slow, discontinous; MF	Atrophy, diffuse leukoencephalopathy	−	Refractory sz; significant ID
	14/F	LGS	0.4	0.2		Sys, IT		BG slow, MF	Atrophy, focal leukoencephalopathy	−	Partially controlled sz; significant ID
	15/F	LGS	3.5	0.7		Sys, IT		BG slow; MF, SSW	Atrophy, diffuse leukoencephalopathy	−	Refractory sz; significant ID
						RT					
	16/F	LGS	10	3.8		Sys, IT		Focal slow; no EDs described	Diffuse leukoencephalopathy		Partially controlled; significant ID
						RT					
	17/F	LGS	0.9	0.3		Sys, IT		No BG description; MF	Diffuse leukoencephalopathy	−	Controlled sz; significant ID
						RT					
	18/M	LGS	3.1	0.5		Sys, IT		BG slow, MF, SSW	Focal leukoencephalopathy	−	Partially controlled sz; significant ID
						RT					
Mitsufuji (1996)^10^	19/M	LGS	9	3		Sys, IT		BG slow, SSW	Multiple ↑T2	−	Refractory sz; progressively worse ID
						RT			Subcortical white matter		
Sawayanagi (1989)^11^	20/	LGS	1.5	0.6		Sys, IT	+	Consistent with LGS	−	−	
						RT					
Akiyama (1983)^12^	21/	LGS	7	3		Sys, IT		Consistent with LGS	−	−	
						RT					

Abbreviations: ↑T2, increased T2 intensity on MRI; A, atonic; AA, atypical absence; ALL, acute lymphoblastic leukemia; ASM, anti‐seizure medications; CC, corpus callosotomy; CNS +, CNS leukemic involvement; F, female; F, focal; G, generalized; GDD, global developmental delay; GSW, generalized spike wave; ID, intellectual disability; IT, intrathecal methotrexate; KD, Ketogenic diet; LGS, Lennox–Gastaut Syndrome; M, male; MF, multifocal; MTX NT, methotrexate neurotoxicity (symptomatic); PFA, paroxysmal fast activity; PSW, polyspike wave; RT, cranial irradiation; Rx, treatment; SSW, slow spike wave; Sys, systemic chemotherapy; Sz, seizure; T, tonic; VNS, Vagal Nerve Stimulator; Y, years.

MTX neurotoxicity, linked to high doses or IT use, can manifest acutely, sub‐acutely, or long‐term.^3^ MTX, a dihydrofolate reductase inhibitor, causes folate deficiency and elevated homocysteine levels, leading to vascular endothelial injury.^2^ Furthermore, homocysteine is an agonist of NMDA (*N*‐methyl‐d‐aspartate) and AMPA (amino‐3‐hydroxy‐5‐methyl‐4‐isoxazolepropionate)/Kainate ionotropic glutamate receptors, resulting in excess brain excitation.^2^ Cranial irradiation and IT MTX are suspected to contribute to EE, with 12 cases reported in the literature.^6^, ^7^, ^8^, ^9^, ^10^ Brain irradiation can exacerbate neurotoxic effects by compromising the blood–brain barrier and enhancing chemotherapy's impact.^10^ However, it is unlikely an integral factor, as none of our cases underwent brain irradiation.

In our series, children were young at diagnosis of ALL, with a mean age of 1.9 years. In one previous case series with EE, the mean age of ALL therapy was <1 year,^9^ and 2 to 3 years in other series.^5^, ^7^, ^8^, ^10^ The young age of ALL is a potential risk factor. Development of aberrant cortical and thalamocortical connectivity in the immature, incompletely myelinated brain is hypothesized in the pathogenesis of LGS, in this instance, instigated by neurotoxicity.^13^

In a large systematic review, 1.3% developed epilepsy, of which half experienced symptomatic MTX neurotoxicity.^3^ However, three of our patients experienced symptomatic MTX neurotoxicity, similar to other reports,^5^ suggesting it may contribute to EE development. MRI findings in EE cases vary and can include normal imaging. In our series, all patients showed mild diffuse atrophy with or without small white matter hyperintensities, but no structural abnormalities to explain seizures. A negative MRI does not rule out neurotoxicity.^2^ While leukoencephalopathy occurs in all symptomatic and 20% of asymptomatic MTX neurotoxicity patients,^2^ the incidence of epilepsy is much lower.^3^ Reduced white matter volumes in ALL survivors are linked to attention and IQ deficits,^2^ but MRI has limited predictive value for multifocal/generalized EE. In contrast, focal DRE cases may develop focal abnormalities like mesial temporal sclerosis after MTX therapy.^4^

Individual predisposition to drug‐resistant EE, with similar ALL treatment regimens is not fully elucidated. All study patients had genetic testing. Case 1 had a 14q32 duplication linked to myeloproliferative disorders and a 16p11.2 deletion associated with language delay. Case 3 had a 17q21.31 missense mutation of unknown significance. None of these mutations are previously linked to EE. Genetic polymorphisms related to neurogenesis may increase the risk of MTX‐related subacute neurotoxicity.^14^ Yet, no studies demonstrate a specific genetic predisposition to chronic toxicity or epilepsy. Notably, 2 adenosine receptor (ADORA2A) polymorphisms, rs2298383 and rs5760410, are associated with MTX adverse effects.^2^ Furthermore, epilepsy studies have shown that alterations of adenosine levels and receptors, including ADORA2A, are linked with neuronal damage and CNS hyperexcitability, leading to abnormal neuronal circuitry and epileptogenesis.^15^ Genetic polymorphism testing was not available for our patients.

Managing seizures in patients with EEs is particularly challenging. One patient tried the ketogenic diet with limited success, similar to two other cases in the literature.^5^, ^7^ Good outcomes with temporal or extra‐temporal resections occur in patients with focal epilepsy,^5^, ^7^ but palliative surgery results are limited. Two of our patients showed minimal response to CC, with daily seizures persisting, similar to 2 other reported patients with CC.^5^, ^7^ Another individual had resolution of atonic seizures, but persistent weekly to monthly seizures after CC.^5^ None of our patients had a vagal nerve stimulator (VNS), although 8 reported VNS p1atients had minimal response, many of whom also had CC.^5^, ^6^, ^7^, ^8^ More data are needed on neuromodulation techniques, like deep brain stimulation. All our patients had severe neuropsychiatric comorbidities requiring close psychiatric management.

Families should be counseled that DRE is rarely associated with successful childhood ALL treatment. The young age of ALL diagnosis (<3 years) may be a predisposing factor. Although focal epilepsy may be amenable to resective surgery, palliative treatments, including VNS, and CC have limited efficacy. Multi‐center studies on epilepsy biomarkers, including advanced genetics, neuroimaging, and electrophysiology, are needed to personalize treatments and prevent epileptogenesis.

## FUNDING INFORMATION

This research received no specific grant from any funding agency in the public, commercial, or not‐for‐profit sectors.

## CONFLICT OF INTEREST STATEMENT

The Authors declare that there is no conflict of interest.

## PATIENT CONSENT

All provided consent for the case to be published.

## Supporting information


Figure S1.
