# Supplementary figures and images for: Developmental and epileptic encephalopathies after successful treatment of pediatric ALL: A case series and review of literature
Source: Epileptic Disord. 2024 Sep 11;26(6):858–67. doi: 10.1002/epd2.20280 (PMC11651377; doi:10.1002/epd2.20280)

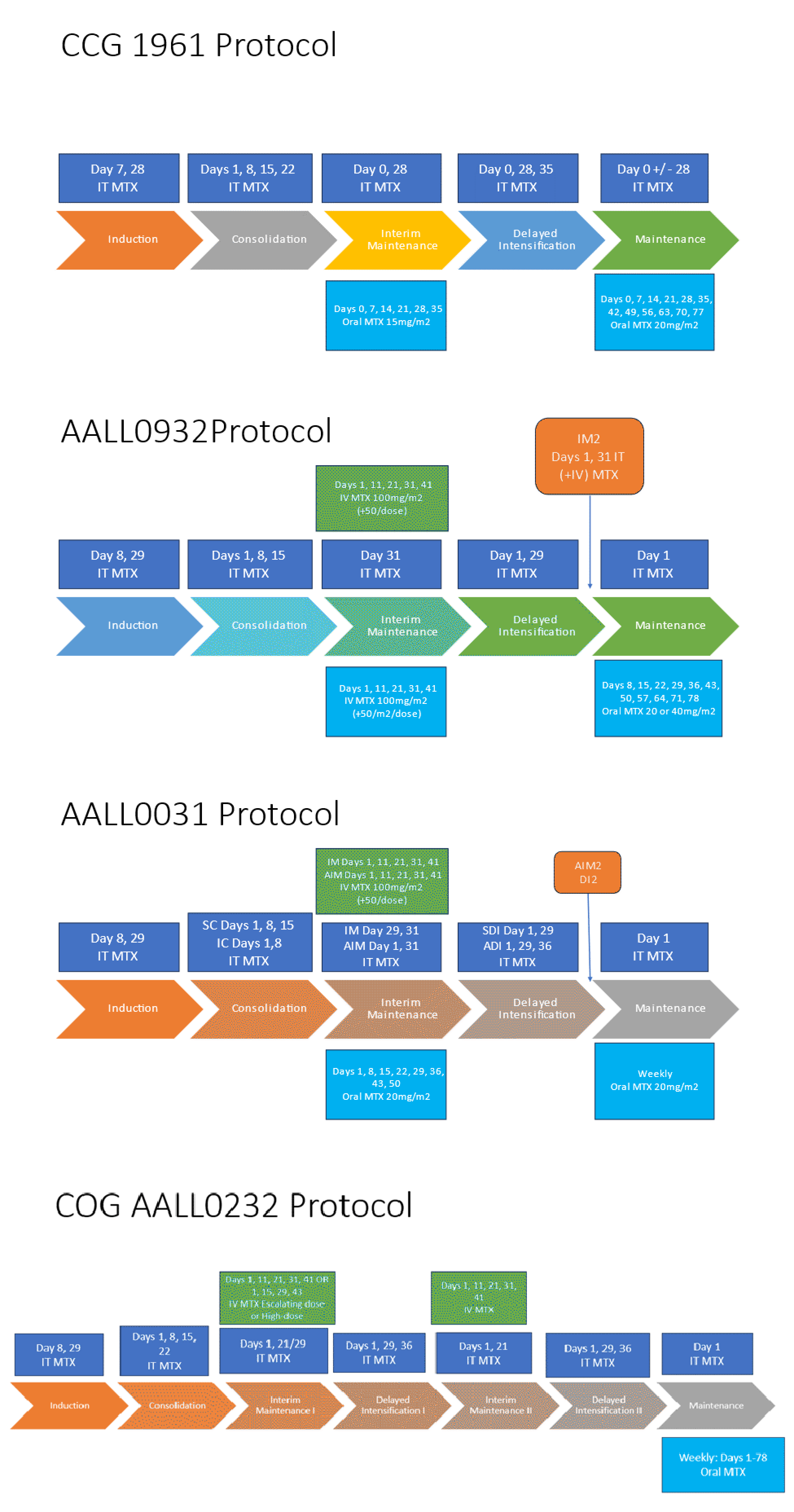

Supplement: Supplementary file 1 — Figure S1. [file EPD2-26-858-s001.tif]
